# Role of inferior vena cava collapsibility index in the prediction of hypotension associated with general anesthesia: an observational study

**DOI:** 10.1186/s12871-019-0809-4

**Published:** 2019-08-07

**Authors:** Marcell Szabó, Anna Bozó, Katalin Darvas, Alexandra Horváth, Zsolt Dániel Iványi

**Affiliations:** 1 1st Department of Surgery, Semmelweis University, Üllői út 78, Budapest, 1082 Hungary; 2 Department of Anesthesiology and Intensive Therapy, Semmelweis University, Üllői út 78B, Budapest, 1082 Hungary

**Keywords:** Anesthesia, Hypotension, Propofol, Vena cava, Inferior, Echocardiography

## Abstract

**Background:**

Intraoperative hypotension increases 30-day mortality and the risks of myocardial injury and acute renal failure. Patients with inadequate volume reserve before the induction of anesthesia are highly exposed. The identification of latent hypovolemia is therefore crucial. Ultrasonographic measurement of the inferior vena cava collapsibility index (IVCCI) is able to detect volume responsiveness in circulatory shock. No current evidence is available regarding whether preoperative measurement of the IVCCI could identify patients at high risk for hypotension associated with general anesthesia.

**Methods:**

A total of 102 patients undergoing elective general surgery under general anesthesia with standardized propofol induction were recruited for this prospective observational study. The IVCCI was measured under spontaneous breathing. A collapsing (IVCCI≧50%) (CI+) and a noncollapsing (CI-) group were formed. Immediate postinduction changes in systolic and mean blood pressure were compared. The performance of the IVCCI as a diagnostic tool for predicting hypotension (systolic pressure < 90 mmHg or a ≥ 30% drop from the baseline) was evaluated by ROC curve analysis.

**Results:**

A total of 83 patients were available for analysis, with 20 in the CI+ and 63 in the CI- group, we excluded 19 previously eligible patients due to inadequate visualization of the IVC (7 cases), lack of adherence to the protocol (8 cases), missing data (2 cases) or change in anesthesiologic management (2 cases). The mean decrease in systolic pressure in the CI+ group was 53.8 ± 15.3 compared to 35.8 ± 18.1 mmHg in CI- patients (*P* = 0.0001). The relative mean arterial pressure change medians were 34.1% (IQR 23.2–43.0%) and 24.2% (IQR 17.2–30.2%), respectively (*P* = 0.0029). The ROC curve analysis for IVCCI showed an AUC of 64.8% (95% CI 52.1–77.5%). The selected 50% level of the IVCCI had a sensitivity of only 45.5% (95% CI 28.1–63.7%), but the specificity was high at 90.0% (78.2–96.7%). The positive predictive value was 75.0% (95% CI 50.9–91.3%), and the negative predictive value was 71.4% (95% CI 58.7–82.1%).

**Conclusion:**

In spontaneously breathing preoperative noncardiac surgical patients, preoperatively detected IVCCI≧50% can predict postinduction hypotension with high specificity but low sensitivity. Despite moderate performance, IVCCI is an easy, noninvasive and attractive option to identify patients at risk and should be explored further.

**Electronic supplementary material:**

The online version of this article (10.1186/s12871-019-0809-4) contains supplementary material, which is available to authorized users.

## Introduction

Maintaining hemodynamic stability is essential for reducing the rate of postoperative complications. Although intraoperative hypotension has no universal definition, it has a serious impact on myocardial injury, acute kidney injury, septic complications [1], the risk of 30-day mortality [2], as well as the risk of one-year mortality in selected patient populations [3] after noncardiac surgery. Prevention of an undesired hypotensive event has a key role in providing patient safety. To date, available prediction models used for estimation of the risk of hypotension are mostly based on nonmodifiable factors (e.g., age, comorbidities) [4, 5]. There is a need to identify easily available variables that can help clinicians recognize patients with a modifiable risk level, such as those with impaired preload.

Hypovolemia is probably the most common factor provoking postinduction hypotension, despite worldwide improvement in preoperative optimization and changing practices promoting the avoidance of unnecessary fasting and mechanical bowel preparation, optimized fluid therapy remains the cornerstone of treatment with excellent effectiveness [5]. The identification of latent hypovolemic patients affords clinicians a chance to implement proper fluid replacement before inducing general anesthesia. Without a universal definition, we consider latent hypovolemia a clinical condition corresponding to a decrease in circulating blood volume without obvious hemodynamic changes and/or organ dysfunction, which increases the risk of the development of hypoperfusion in response to external impacts such as anesthesia and surgery.

Several invasive devices (e.g., pulmonary arterial catheter, PiCCO®, Vigileo®, etc.) are available for evaluating preload among other elements of hemodynamic status, but their universal use is not a reasonable option due to financial constraints, relatively high complication rates, known limitations and unnecessary invasiveness compared to most surgical procedures [6].

The use of noninvasive ultrasound examination by anesthesiologists is a widespread and useful aid in the safe application of anesthesia. In a recent meta-analysis, Ferreira et al. reported an approximately 31% change in anesthesia management when ultrasound was used. Thirty-five percent of the performed ultrasonographies were transthoracic echocardiographies [7]. A standard transthoracic echocardiography revealing all relevant cardiologic details takes a significant amount of time, even when performed by adequately trained cardiologists; however, focused goal-directed scans are much faster while maintaining important clinical relevance [8]. The proper training of anesthesiologists is an issue because all types of sonographies are operator-dependent exams. However, a French center reported that parameters of high anesthesiological importance such as global left ventricular function, ventricular diameters, pericardial effusion or the diameter of the inferior vena cava were adequately evaluated by trainees who had taken part in a 12 h learning program [9]. Concerning volemic status, the variability of the diameter of the inferior vena cava (IVC), which follows the respiratory cycle, is considered to be a valuable predictor of volume responsiveness in cases of circulatory failure in ventilated [10–13] and spontaneously breathing patients [14, 15] even in the presence of nonfatal cardiac arrhythmias [16]. In these studies, clinically evident volume responsiveness was defined as an at least 10% increase in cardiac output in response to bolus fluid administration.

In the present study, we aimed to characterize the collapsibility index of the IVC as a potential screening tool to identify patients who were candidate for hypotensive events related to general anesthesia in an otherwise hemodynamically stable population.

## Materials and methods

### Patients

This prospective, observational study was conducted between 26/07/2016 and 30/10/2018 in the 1st Department of Surgery, Semmelweis University, Budapest, Hungary. Ethics approval for this study was provided by Semmelweis University Regional and Institutional Committee of Science and Research Ethics, Budapest, Hungary (Registration number: 144/2016, date of approval: 25/07/2016). Informed consent was obtained from each subject. Patients aged ≥18 years who were scheduled for elective general surgery under general anesthesia on predetermined weekdays were included if they met the eligibility criteria and all necessary data were available. The inclusion and exclusion criteria are shown in Table 1. As a conceptual summary, we included elective, premedicated patients and excluded those who were already hypotensive or severely hypertensive, those considered to be at high risk or those having a clinical condition that would prevent the adequate evaluation of either the IVC (e.g., significant tricuspid regurgitation) or blood pressure changes (e.g., pheochromocytoma).

**Table 1:** Inclusion and exclusion criteria

Inclusion criteria	Exclusion criteria
Age ≥ 18 years Elective surgery General anesthesia	ASA physical status > 3 Dyspnea Systolic blood pressure ≥ 180 mmHg Systolic blood pressure < 90 mmHg Decompensated heart failure Elevated pulmonary arterial pressure > 40 mmHg Significant valvular disease Significant carotid stenosis Documented negative fluid balance > 1.000 ml on preceding day Pheochromocytoma SOFA score > 1 Agitation (RASS > 1) IVC non visualized Epidural catheter in use

*ASA* American Society of Anesthesiology, *IVC* inferior vena cava, *RASS* Richmond Agitation Sedation Scale, *SOFA* Sepsis-related Organ Failure Assessment

### Study design

Eligible patients were screened using ultrasonography. The inferior vena cava was identified, and characteristics were recorded in the dorsal recumbent position under light sedation (RASS 0- -1) and spontaneous breathing. The collapsibility index (IVCCI) was calculated, and two groups were formed according to the measured IVCCI: the collapsing group characterized by IVCCI≥50% (CI+) and the noncollapsing group (CI-) (in whom the IVCCI was< 50%). This level was arbitrarily set with regard to the results in previously published literature, verifying that IVCCI values between 40 and 50% measured in spontaneously breathing patients are predictive for volume responsiveness in different clinical settings [14–16].

Vital signs were recorded, and protocolled anesthesia induction was performed. Two minutes after drug administration but before intubating the trachea, the vital signs were measured again. The hemodynamic response was characterized in each group with the change in systolic blood pressure as the end point. Anesthesia-related hypotensive events were recorded if the systolic blood pressure dropped below 90 mmHg or a ≥ 30% drop in initial systolic pressure was observed.

### Ultrasonographic measurements

**Fig. 1:**
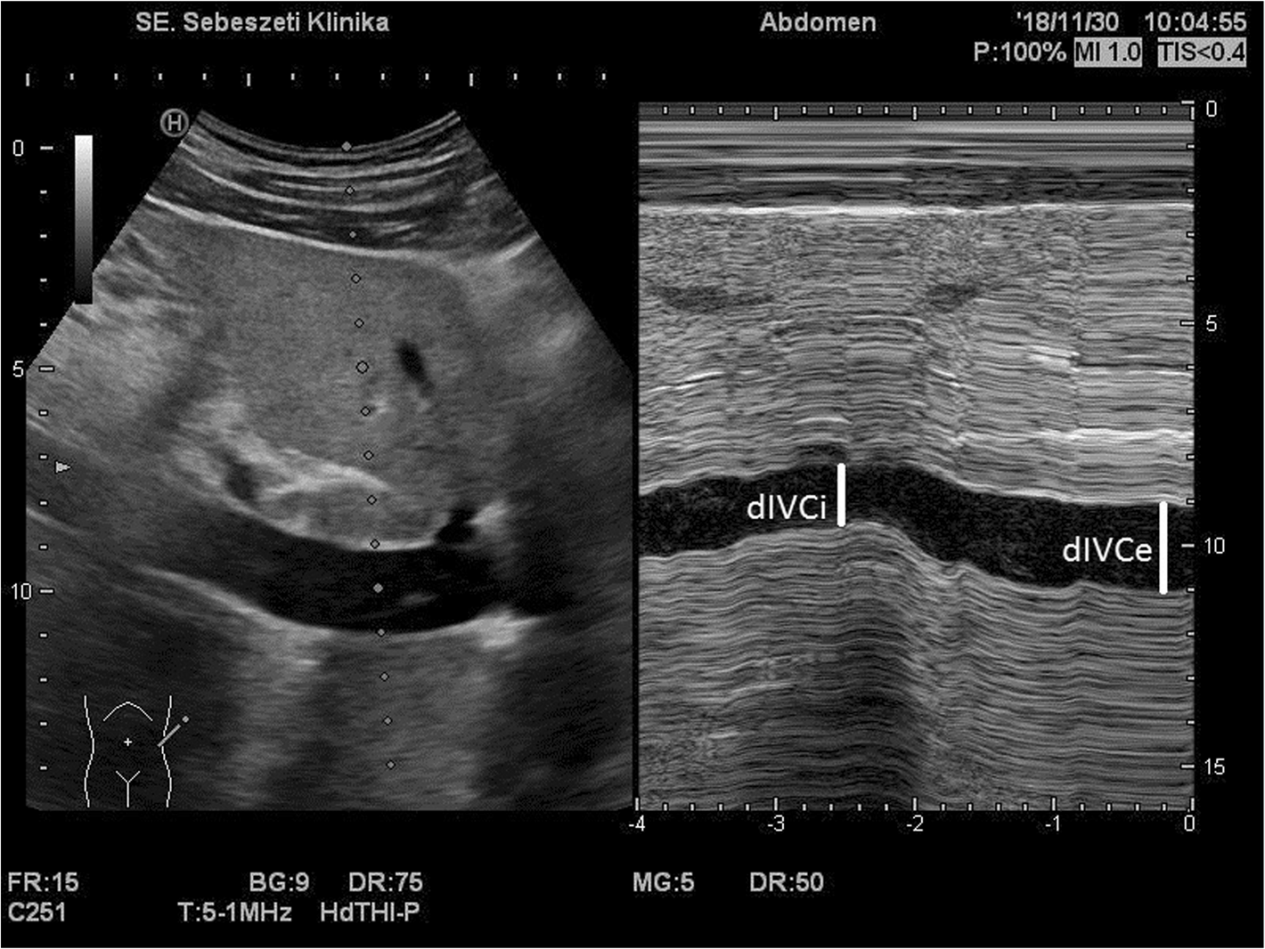
Typical ultrasound image of the inferior vena cava near the heart. M-mode image represents high respiratory collapsibility. (dIVCe = IVC diameter in expiration, dIVCi = IVC diameter in inspiration)

Patients in the surgical ward were evaluated before transportation to the operating room. Ultrasonographic scans were performed by one of four adequately trained independent anesthesiologists who had undergone institutional training for ultrasound use in anesthesia and who had at least 2 years of experience in the field. One of two ultrasound machines was used (Sonosite Titan - FUJIFILM SonoSite, Inc. Bothell, Washington, United States and Hitachi Aloka Noblus, Hitachi Healthcare, Tokyo, Japan). Both machines were equipped with a curvilinear transducer (5 MHz). The inferior vena cava was visualized in B-mode from a longitudinal paramedian subxyphoid view; when a good echographic window was not available, an intercostal, transhepatic lateral view was used. The last section of the vein, which was proximal to the hepatic vein inflow and 0.5–3 cm from the right atrium, was selected for the M-mode, and measurements were performed as recommended in the consensus document of the American and European Cardiologic Societies [17]. The maximal expiratory diameter of the vein was recorded (dIVC expiration) under normal breathing of the lightly sedated patient, and the collapsibility index (IVCCI) was calculated using the following formula: (dIVC expiration – dIVC inspiration) / dIVC expiration × 100 = IVCCI. The IVC diameter at expiration and inspiration had to be measured during the same respiratory cycle. Figure 1 represents a typical highly collapsing IVC.

#### Anesthesiologic practice

Routine premedication using alprazolam was given 1 hour before surgery. Regular cardiovascular medication of the patients was maintained on their established routine, except for diuretics and angiotensin-converting inhibitors, which were withdrawn. All patients were monitored continuously using ECG, pulse oximetry and capnography starting from the beginning of manual ventilation. Noninvasive blood pressure monitoring by oscillometry and invasive arterial blood pressure monitoring were used at the discretion of the anesthesiologist according to the details of the planned surgery and the risk level of the patient. Noninvasive measurements were obtained at 5 min intervals, and an additional measurement was obligatory 2 min after induction drug administration. This step preceded the intubation of the trachea. If invasive monitoring was used, an arterial cannula was inserted before induction, and postinduction vital signs were registered at the same time points as above. To induce general anesthesia, our institutional standard practice of using fentanyl (1–2 μg/kg), propofol (1,5–2 mg/kg) and nondepolarizing muscle relaxants (rocuronium or cis-atracurium) according to age, weight, chronic organ function and the needs of the surgery was not changed for study purposes.

### Statistical analysis

#### Sample size

To calculate the sample size, the change in systolic blood pressure after induction drug administration was the variable of interest. A minimum difference of 15 mmHg was considered clinically important, and that in combination with a standard deviation of 25 mmHg coming from our pilot data of 103 patients not involved in the study were used for the calculations. A type one error of 0.05 and a required power of 0.80 were set. Assuming unequal study groups with a 1 to 3 ratio of patients having collapsing (CI+) and noncollapsing (CI-) IVC, we used corrected sample sizes [18]. A minimum of 81 patients were required based on the conditions detailed above. To maintain adequate power in cases of a lack of adherence to the protocol or methodological failure, an additional 25% was screened, and a total of 102 patients were enrolled.

#### Data analysis

Data were pooled for analysis in Microsoft Excel 2013 (Additional file 1); for the statistical analysis, we used StatsDirect Statistical Software (Version 3.1.20, Stats Direct Ltd., Grantchester, Cambridge, UK). Continuous variables are presented as the means±standard deviation if they were normally distributed as tested by the Shapiro-Wilk W test. Nonnormally distributed data are shown as the medians and interquartile ranges. Student’s two-sample t-test and the Mann-Whitney U test were used for comparisons. Categorical variables are shown as percentages and absolute numbers of cases. The χ^2^ and Fisher exact test were used for contingency table analysis as appropriate. Two-sided *p*-values are shown, and the limit of statistical significance was set to *p* < 0.05.

The 50% value of IVCCI as a diagnostic cutoff value was evaluated by calculating the sensitivity, specificity, and positive and negative predictive values. Previously cited literature [14] data highlight the potential cutoff level for IVCCI of 40%; this value was also tested. The receiver operating characteristics curve was plotted, and the area under the curve was calculated by Wilcoxon’s method, and the standard error was calculated according to the method by DeLong. In these calculations, a composite definition of hypotension was used. Postinduction systolic pressure less than 90 mmHg and/or a more than 30% decrease from the baseline systolic pressure was needed to treat data as positive for hypotension.

## Results

### Population demographics and characteristics

A total of 102 patients were recruited. We had to exclude 19 previously eligible patients due to inadequately visualized IVC (7 cases), a lack of adherence to the protocol (8 cases), a lack of data (2 cases) or a change in anesthesiological management (2 cases). Finally, 83 patients who matched all inclusion criteria were enrolled. Twenty patients were evaluated in the CI+ group and 63 in the CI+ group. The list of surgical operations performed is provided in Table 2. Baseline characteristics in terms of anthropometry, physiologic status, comorbidities and important prescreening treatments are summarized in Table 3. All variables except age were similar in the two groups without any significant intergroup differences. The median age in the CI+ group was 8 years older than that in the CI- group.

**Table 2:** Surgical procedures in the CI+ and CI- groups

Surgery types	Collapsible (CI+) group (*N* = 20)	Noncollapsible (CI-) group (*N* = 63)	*P* value
Minor procedures	30.0%	34.9%	0.6851
minor laparoscopies, N	2	8	
hernia repairs, N	1	7	
breast and plastic surgeries, N	1	3	
minor perianal procedures, N	0	2	
endocrine surgeries, N	1	2	
Major procedures	70.0%	65.1%	
upper gastrointestinal, N	2	5	
hepatic resections, N	2	4	
pancreatic-biliary surgeries, N	3	10	
colorectal	7	20	
other intestinal	1	2

**Table 3:** Baseline population characteristics

Variable	Collapsible (CI+) group (*N* = 20)	Noncollapsible (CI-) group (*N* = 63)	*P* value
Age, years, median (IQR)	69 (60.5–77)	61 (51–82)	0.0066
Male sex, N (%)	7 (35.0%)	29 (46.0%)	0.3858
BMI, kg/m^2^	24.15 ± 3.04	26.48 ± 4.94	0.0505
ASA 3, N (%)	9 (45.0%)	16 (25.4%)	0.0959
COPD, N (%)	2 (10.0%)	7 (11.1%)	0.9999
Hypertension, N (%)	14 (70.0%)	36 (57.1%)	0.3060
Peripheral arterial disease, N (%)	3 (15.0%)	4 (6.4%)	0.3510
Diabetes, any type, N (%)	4 (20.0%)	12 (19.1%)	0.9999
Preoperative fluid intake, ml (IQR)	700 (500–1400)	600 (100–1200)	0.1438
Baseline systolic pressure, mmHg	147 ± 16	143 ± 17	0.3218
IVC diameter in expiration, mm	17 ± 3	18 ± 4	0.2031
Propofol dose for induction, mg/kg	1.77 ± 0.15	1.81 ± 0.16	0.3756

*ASA* American Society of Anesthesiology, *BMI* body mass index, *COPD* chronic obstructive pulmonary disease, *IQR* interquartile range, *IVC* inferior vena cava

### Hemodynamic data

**Fig. 2:**
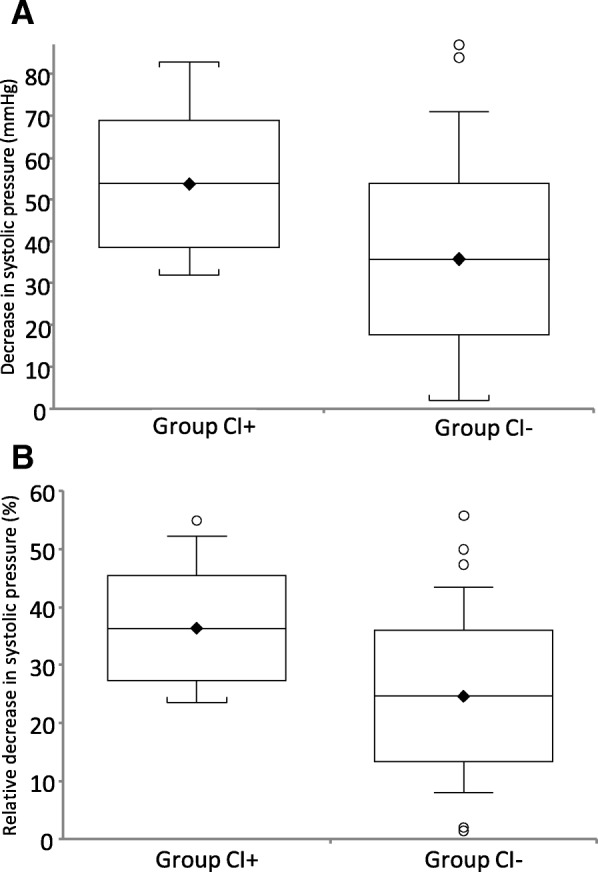
Decrease in systolic pressure after the induction of anesthesia measured in the study groups. **a** absolute decrease in mmHg (mean, standard deviation and range). **b** relative decrease (percentage) from the baseline (mean, standard deviation and range). For group definitions, see the text

The mean postinduction decrease in systolic pressure in the CI+ group was 53.8 ± 15.3 mmHg, which was significantly higher than the 35.8 ± 18.1 mmHg observed among CI- patients (*P* = 0.0001). The same phenomenon was present in the case of a relative decrease in systolic pressures: CI+ patients had a mean of 36.4 ± 9.1%, while this was 24.7 ± 11.3% in the CI- group (*P* < 0.0001). The results are shown in Fig. 2a (absolute decrease) and 2b (decrease relative to the preinduction level). Similarly, the relative MAP change medians were 34.1% (IQR 23.2–43.0%) and 24.2% (17.2–30.2%) (*P* = 0.0029) (data not graphed). Meanwhile, no difference was detectable in heart rate decrease, as CI+ patients presented a median of only 4 bpm (0 bpm–9.5 bpm), and 1 bpm (− 3 bpm–7 bpm) was observed among CI- patients (*P* = 0.1901).

### Performance characteristics of IVC collapsibility

**Fig. 3:**
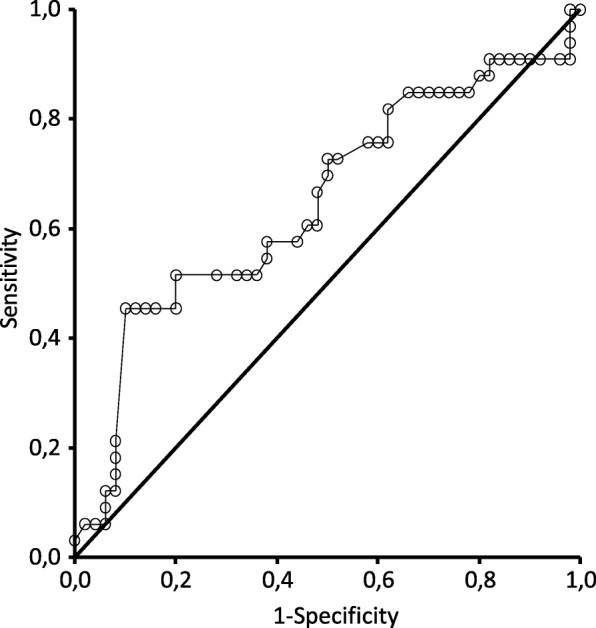
Receiver operating characteristics (ROC) curve of IVC collapsibility for the prediction of hypotension

The ROC curve analysis of the ability of the IVCCI to predict hypotension after the induction of general anesthesia resulted in an AUC of 64.8% (95% CI 52.1–77.5%), demonstrating a poor to fair diagnostic accuracy (Fig. 3). As we previously and arbitrarily set our cutoff value of IVCCI to 50% to distinguish between the CI+ and CI- groups, this particular value was evaluated. The sensitivity was only 45.5% (95% CI 28.1–63.7%), but the specificity was as high as 90.0% (95% CI 78.2–96.7%), giving a positive likelihood ratio of 4.5 (95% CI 1.8–11.3). The positive predictive value was 75.0% (95% CI 50.9–91.3%), and the negative predictive value was 71.4% (95% CI 58.7–82.1%). The IVCCI cutoff of 40% was less promising. The sensitivity became 51.5% (95% CI 33.5–69.2%), and the specificity was 72.0% (95% CI 57.5–83.8%), with positive and negative predictive values of 54.8% (95% CI 36.0–72.7%) and 69.2% (95% CI 54.9 to 81.3%), respectively. In this case, the positive likelihood ratio was only 1.84 (95% CI 1.06–3.20). Setting the cutoff value to 50% was supported by the ROC curve’s Youden index, indicating this value is the optimal cutoff value.

## Discussion

Our prospective observational study compared the hemodynamic consequences of the induction of general anesthesia by propofol in patient groups defined on the basis of having collapsing (IVCCI ≥50%) or noncollapsing (IVCCI< 50%) IVC, and we evaluated the diagnostic value of a high IVCCI value in the prediction of postinduction hypotension. We detected important differences between the two groups, verifying that a high IVCCI value was associated with a more profound change in systolic blood pressure and mean arterial pressure. This remarkable phenomenon is consistent with the general concept that the IVCCI is able to detect volemic changes. The IVCCI had high specificity and low sensitivity in experiments by Mueller et al. when a cutoff level of > 40% was used to predict volume responsiveness [14]. However, this performance as a diagnostic tool is, on the one hand, similar to that observed in our experiments in the case of the value of 50%, but on the other hand, the physiologic meaning is different: we consider values indicating high collapsibility measured in normotensive or even hypertensive patients as a sign of latent volume depletion when general anesthesia and the consequent deactivation of the sympathetic compensatory mechanisms might have profound effects.

Of note, most previous data are from intensive care settings in which IVC diameter and the IVCCI were used to identify volume-responsive patients in circulatory shock [10–16]. Our approach has several new aspects. First, although it is not entirely unique, we performed IVC measurements in a general anesthesiologic setting. Such studies are still controversial in spinal anesthesia, in which sympathetic denervation could also reveal inadequate fluid reserve. Mačiulienė et al. failed to detect a prognostic role of the IVCCI [19], while another recent trial reported an IVCCI-based fluid replacement strategy as a useful tool to reduce the incidence of hypotension [20].

Second, contrary to the results of the abovementioned articles, the IVCCI was evaluated in an otherwise hemodynamically stable patient population. Despite our systematic screening of the relevant literature, we were not aware of a similar study at the preparation of our study protocol. However, Zhang et al. reported the use of the IVCCI for the prediction of hypotension attributable to general anesthesia [21]. Some important differences must be emphasized here. An important proportion of their patients came from a cardiac surgical population, which probably contributed to the choice of etomidate as an intravenous anesthetic. The more prominent hypotension frequency (42/90) in that study compared to ours (33/83) despite the relatively safe agent is important and most likely attributable, at least in part, to the high proportion of cardiac surgical patients. Our choice of propofol comes from its widespread use for everyday purposes and consequently high variability of the affected population. This choice is Janus-faced, as propofol has a well-known potential to provoke hypotension itself by several mechanisms, the most important of which is vasodilation [22, 23], making the agent an independent risk factor for hypotension [24], but the protocolized anesthetic strategy, provided to all the enrolled patients, excludes the induction agent as an independent risk factor.

Our observational trial has limitations. First, we had to exclude 19 patients despite a slightly better visualization rate compared to that in the previously cited study with a similar approach [21], but adherence to the protocol was lower than optimal. The final population was still large enough to not jeopardize our statistical power. Second, spontaneous breathing is difficult to standardize, and we hypothesized that premedicated patients without obvious signs of agitation (RASS≤1) and respiratory efforts constitute an adequate setting for IVC measurements. Our protocol tends to follow physiologic airway pressure changes but therefore differs from that of some cited studies, which chose forced deep inspiration [15] or sniffing [14, 16]. Additionally, our study, by its observational nature, is not entirely free from intergroup differences: patients in the CI+ group were slightly older than those in the CI- group (median 69 vs 61 years.). Despite the statistical significance, both groups represent a population otherwise highly susceptible (older than 50 years) to hypotension [24]. As an older population is generally more susceptible to profound volume disturbances, we consider this finding a pathophysiologic consequence and not a biasing factor. This feature also characterized the hypotensive population in the study by Zhang et al. [21], and the randomization process allowed Ceruti et al. to characterize their IVCCI-based fluid replacement strategy without this confounder [20].

When we focus on IVCCI’s diagnostic performance, our AUC of 64.8% together with the sensitivity and specificity levels should be evaluated in the context of the models currently available for the prediction of hypotension associated with general anesthesia. A recent multicenter observational study identified age, degree of high blood pressure prior to surgery and type II diabetes as risk factors [5]. As these results, which were similar to former findings [24], are hard to use to stratify individual patients’ risk in everyday practice, a simplified scoring system was also developed by Cheung et al. [4]. In their ‘HEART Score’ model in which preexisting hypotension or bradycardia, elderly age, preoperative renin-angiotensin blockade, revised cardiac risk index (≥3 points), and type of surgery (major surgery) were valuable risk factors, the ROC analysis showed moderate usefulness (AUC 75%). As our protocol ruled out unstable patients, and ACE inhibitors were withdrawn, we consider that IVCCI measurement can reveal additional patients at risk who were potentially not identified by the abovementioned factors. It is important to emphasize that, in contrast to previous models, our observations point to a potentially modifiable variable.

## Conclusion

As a clinical interpretation of our results in spontaneously breathing preoperative noncardiac surgical patients, preoperative IVCCI measurement is feasible and can predict postinduction hypotension with high specificity but low sensitivity. Despite its moderate performance, IVCCI is an easy, noninvasive and attractive option to identify patients at risk of postinduction hypotension and should be explored further. The potential involvement of the IVCCI in multifactorial models can be a field of future studies.

## Additional file

Additional file 1: Dataset of the study. Categorical questions were marked with 1 (yes) or 0 (no). (XLSX 26 kb)

## Data Availability

All data generated or analyzed during this study are included in this published article [and its additional files].

## Abbreviations

ASA: American Society of Anesthesiology
CI: Collapsibility Index
IQR: Interquartile range
IVC: Inferior vena cava
IVCCI: Inferior vena cava collapsibility index
MAP: Mean arterial pressure
RASS: Richmond Agitation Sedation Scale
ROC: Receiver Operating Characteristics
SOFA: Sepsis-related Organ Failure Assessment
